# Adjuvant Ciprofloxacin for Persistent BK Polyomavirus Infection in Kidney Transplant Recipients

**DOI:** 10.1155/2014/107459

**Published:** 2014-05-08

**Authors:** David Arroyo, Sindhu Chandran, Parsia A. Vagefi, David Wojciechowski

**Affiliations:** ^1^Nephrology Department, Hospital General Universitario Gregorio Marañón, C/Doctor Esquerdo 46, 28007 Madrid, Spain; ^2^Kidney Transplant Service, Division of Nephrology, Department of Medicine, University of California, San Francisco, 400 Parnassus Avenue, Suite A701, San Francisco, CA 94143, USA; ^3^Transplant Surgery, Massachusetts General Hospital/Harvard Medical School, 55 Fruit Street, White 544, Boston, MA 02114, USA

## Abstract

*Background*. BK virus (BKV) infection is a common complication following kidney transplantation. Immunosuppression reduction is the cornerstone of treatment while adjuvant drugs have been tried in small uncontrolled studies. We sought to examine our center's experience with the use of ciprofloxacin in patients with persistent BKV infection. *Methods*. Retrospective evaluation of the effect of a 30-day ciprofloxacin course (250 mg twice daily) on BKV infection in kidney transplant recipients who had been diagnosed with BK viruria ≥106 copies/mL and viremia ≥500 copies/mL and in whom the infection did not resolve after immunosuppression reduction and/or treatment with other adjuvant agents. BKV in plasma and urine was evaluated after 3 months following treatment with ciprofloxacin. * Results*. Nine kidney transplant recipients received ciprofloxacin at a median of 130 days following the initial reduction in immunosuppression. Three patients showed complete viral clearance and another 3 had a ≥50% decrease in plasma viral load. No serious adverse events secondary to ciprofloxacin were reported and no grafts were lost due to BKV up to 1 year after treatment. *Conclusion.* Ciprofloxacin may be a useful therapy for persistent BKV infection despite conventional treatment. Randomized trials are required to evaluate the potential benefit of this adjuvant therapy.

## 1. Introduction

BK polyomavirus (BKV) is a common pathogen of kidney transplant recipients, which can result in impaired graft function and inferior graft survival [1]. Approximately 30%, 11–13%, and 8% of kidney transplant recipients develop BK viruria, viremia, and BK virus associated nephropathy (BKVAN), respectively [1–3]. Graft loss rates have been reported to be as high as 30–50% following a diagnosis of BKVAN [1, 4], although more recent data indicate that with early diagnosis of BK viremia or viruria using regular screening, the majority of patients respond favorably [5]. However, BKV infection continues to have a major impact on graft function with nearly 25% of infected patients showing a sustained increase in serum creatinine of at least 50% compared to that observed at the time of diagnosis [6]. Several donor and recipient risk factors for the development of BKV infection have been identified and of these, a high burden of immunosuppression appears to be the most important [7].

A strategy of immunosuppression reduction is therefore considered the cornerstone of treatment for BKV infection [8], although a uniform standardized protocol has not yet been established [2, 9]. The inability to clear this virus despite a reduction in immunosuppression has led to a variety of additional agents with possible anti-BK activity being utilized as adjuvant therapy including leflunomide, cidofovir, and intravenous immunoglobulin (IVIG) [10]. All of these agents have been reported to be beneficial in anecdotal cases. However, the use of these agents has been mostly limited by pharmacologic and logistical issues. IVIG is expensive, potentially nephrotoxic, and needs to be administered intravenously over several hours. Leflunomide has significant hematologic and hepatic toxicities and requires therapeutic drug monitoring due to variable interpatient pharmacokinetics, while cidofovir is highly nephrotoxic and appears to have minimal* in vitro* antiviral activity against BKV.

Fluoroquinolones have been reported to display anti-BKV properties through inhibition of the large T antigen (Tag) helicase activity [11].* In vitro*, fluoroquinolones not only reduce BKV DNA replication and the associated expression of viral proteins such as Tag but also lower the cell release of viral progeny by more than 90% [12]. Several studies have explored the efficacy of fluoroquinolones for the prevention and treatment of BKV-associated hemorrhagic cystitis after hematopoietic stem cell transplantation, which they found to be reasonably successful [13, 14]. On the other hand, a recently published multicenter, randomized, prospective trial utilizing a 30-day course of levofloxacin compared to placebo in kidney transplant recipients for the treatment of BK viremia found no improvement in BK viral load reduction or renal allograft function [15]. However, in this study the immunosuppression reduction was not standardized and all patients had newly diagnosed BK viremia.

Given the accumulated evidence in the literature indicating* in vitro *activity of fluoroquinolones against BKV and the mixed data currently available on their clinical efficacy for the treatment of BKV infection, we decided to examine our own experience. The aim of our study was to retrospectively evaluate the effect of adding a 30-day course of ciprofloxacin to the treatment of persistent BKV infection in kidney transplant recipients who were refractory to immunosuppression reduction and treatment with other adjuvant agents.

## 2. Methods

### 2.1. Study Design and Patient Population

This is a single-center retrospective study of kidney transplant recipients with refractory BKV infection who received adjuvant therapy with ciprofloxacin 250 mg orally twice daily for 30 days. Patients were identified through a search of an electronic database of transplant recipients at UCSF Medical Center and had received a kidney transplant between July 2009 and June 2010. This time frame was selected as we collected information during those dates in a central database on all UCSF transplant recipients to evaluate our center specific BKV outcomes and treatment strategies. Refractory BKV infection was defined as persistently detectable viremia (≥500 copies/mL) and high grade viruria (≥10^6^ copies/mL) despite immunosuppression reduction and the use of other adjuvant agents. This study was approved by our institutional review board as a retrospective analysis and informed consent was therefore not required. Outpatient and inpatient medical records were reviewed, including clinic visit notes, hospital notes, discharge summaries, and medication histories. Data obtained included patient demographics, transplant characteristics, laboratory and pathology results, and BKV urine and plasma PCR. The primary end-point was BKV plasma clearance, defined as an undetectable plasma viral load, 3 months after the initiation of ciprofloxacin. Secondary end-points included the percent reduction in BK viremia and the number of patients with >50% reduction in BK viremia 3 months after the initiation of ciprofloxacin as well as renal allograft function 3, 6, 9, and 12 months after the initiation of ciprofloxacin.

### 2.2. BKV Monitoring

The BKV screening protocol at our institution consists of monitoring quantitative BKV DNA PCR in urine and plasma at 1, 3, 6, 9, and 12 months after transplantation using the Focus 3M Integrated Cycler. Screening is considered positive at BK viruria ≥10^6^ copies/mL and/or viremia ≥500 copies/mL. The diagnosis of BKVAN is made by a kidney allograft biopsy showing positive immunohistochemical staining for the SV-40 large T antigen with or without findings consistent with viral-mediated tubular epithelial cell damage and interstitial inflammation. Biopsies are performed at 6 months after transplantation for surveillance and at any other time for clinical indications such as elevated serum creatinine or persistently high BK viremia despite therapeutic interventions.

### 2.3. BKV Management

Our initial approach to BKV infection consists of a reduction in immunosuppression. The first step is a 50% decrease in the dose of the antimetabolite drug. Monthly BKV monitoring is performed until infection resolution, defined as undetectable BK viremia. If this goal is not achieved within 1–3 months, physicians may choose to further lower or discontinue the antimetabolite, lower the calcineurin inhibitor (CNI) trough goal, or switch from tacrolimus to cyclosporine. If a second round of immunosuppression reduction is not effective, adjuvant treatment with IVIG, cidofovir, leflunomide, and/or ciprofloxacin is considered.

## 3. Results

We identified 9 kidney transplant recipients with persistent BKV infection despite immunosuppression reduction, who subsequently received a 30-day course of ciprofloxacin. Baseline demographic and transplant characteristics of these recipients are summarized in Table 1. There were 5 men and 4 women. Seven received their kidney transplant from a deceased donor. Only 1 patient received a ureteral stent. All received antibody induction: rabbit thymoglobulin in 3 patients and basiliximab in 6 patients. Initial maintenance immunosuppression consisted of a CNI, mycophenolate mofetil (MMF), and prednisone in 7 of the 9 patients while the remaining 2 patients underwent early corticosteroid withdrawal and were maintained on only a CNI and MMF. One patient experienced an episode of antibody-mediated rejection which occurred prior to the BKV infection.

Median time to the first screen positivity of BKV infection was 95 days posttransplantation (range 31 to 224 days). Serum creatinine at the time of diagnosis ranged between 0.54 and 2.12 mg/dL. All patients had high-grade viruria and viremia at diagnosis. Management of immunosuppression and use of other antiviral therapies are summarized in Table 2. MMF had been reduced in all patients and then withdrawn completely in 6 of them. Leflunomide therapy replaced MMF in two of those in whom MMF was discontinued. Eight patients had received cidofovir and 4 had received IVIG prior to ciprofloxacin. Ciprofloxacin 250 mg twice daily for 30 days was added to the other treatments at the physician's discretion at a median of 130 days (range 59 to 379 days) following the diagnosis of BKV infection. All patients had persistent BK viremia at the time of initiation of ciprofloxacin. From the initial diagnosis of BK viremia to the start of ciprofloxacin, BK viremia had improved in 5 patients, worsened in 2 patients, and was unchanged in 2 patients.

All 9 patients underwent a renal allograft biopsy for cause after the BKV infection was diagnosed, and this demonstrated BKVAN in 7 of them. Ciprofloxacin was given before the diagnosis of BKVAN in 4 patients (range: 9–61 days; median 48 days) and after the diagnosis of BKVAN in 3 patients (range: 25–93 days; median 45 days). Three patients demonstrated complete resolution of viremia and another 3 had a decrease of more than 50% in plasma viral load at 3 months following the initiation of ciprofloxacin treatment (Table 3). Of the remaining 3 patients, 2 had an increase in the plasma viral load and 1 had a decrease of <50% at 3 months. The average reduction in viral load from the initiation of ciprofloxacin to month 3 was 58.8%. A higher percentage of patients who had a lower level of BK viremia from the time of initial diagnosis to starting ciprofloxacin had more than 50% reduction of BK viremia 3 months after ciprofloxacin (100%) compared to those patients with the same or higher level of BK viremia at the start of ciprofloxacin compared to the time of the initial diagnosis (25%; *P* = 0.48).

**Figure 1:**
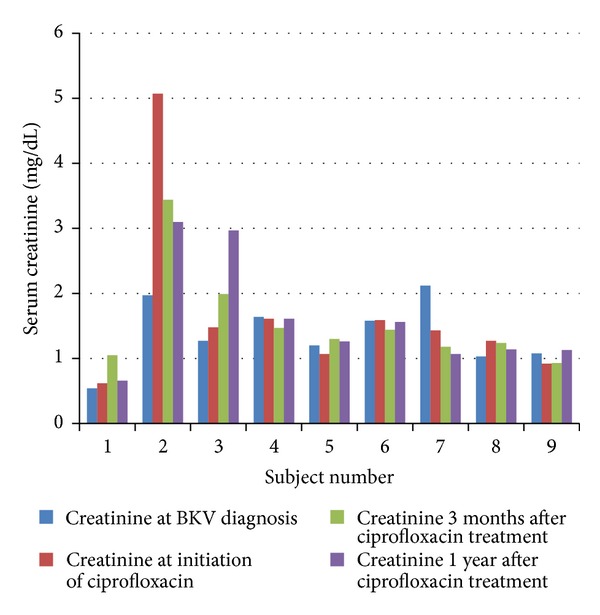
Change in serum creatinine from initial BKV diagnosis to 30 days after ciprofloxacin.

Overall, renal function remained relatively stable or improved in most patients after ciprofloxacin treatment with a mean serum creatinine of 1.67 ± 1.32 mg/dL at the initiation of ciprofloxacin compared to 1.61 ± 0.85 mg/dL one year after ciprofloxacin treatment (*P* = 0.84). However, serum creatinine rose significantly in patient 3 from 1.48 to 2.97 mg/dL (Figure 1). No allografts were lost due to BKV infection up to 1 year after the initiation of ciprofloxacin. There were no cases of tendonitis or tendon rupture,* Clostridium difficile* colitis, or ciprofloxacin-resistant infections during the ciprofloxacin treatment and assessment periods.

## 4. Discussion

BKV is a well-recognized infection in kidney transplant recipients, with known adverse effects on graft function and long-term graft survival. There is general consensus that immunosuppression reduction is the first line treatment for newly diagnosed BKV infection [8]. On the other hand, despite multiple published reports of different adjuvant therapies for refractory cases, a clear advantage of one drug or agent has yet to be demonstrated. In this report, we have described our experience with a novel treatment approach utilizing adjuvant ciprofloxacin for the management of persistent BKV infection in kidney transplant recipients who have failed to resolve the infection with alternative measures.

Our report shows that in a majority (67%) of patients with persistent BKV infection despite immunosuppression reduction, there was either a significant reduction or complete resolution of BK viremia after the addition of ciprofloxacin. At the same time, no adverse events of therapy such as tendonitis or* Clostridium difficile* colitis were noted. Additionally, no cases of fluoroquinolone-resistant urinary tract infections occurred in the follow-up period. Overall, renal function remained stable and no renal allografts were lost due to BKV infection within 1 year of the ciprofloxacin treatment. Ciprofloxacin treatment was well tolerated without any serious side effects in our small group of patients. Clinically, the fluoroquinolone antibiotics represent a convenient and relatively inexpensive class of medications that could treat persistent BKV infections with a low incidence of adverse events.

We readily acknowledge the limitations of our study. This is a descriptive case series with a small number of patients and retrospective data analysis. Since ciprofloxacin was used only in patients with persistent BKV infection, we could not examine the effect of this treatment as a* de novo* early therapy.* In vitro* evidence of the inhibitory effect of fluoroquinolones on BK viral replication has been previously demonstrated [11, 16]. Several studies have explored the efficacy of fluoroquinolones for the prevention and treatment of BKV-associated hemorrhagic cystitis after hematopoietic stem cell transplantation and found fluoroquinolones to be reasonably successful [13, 14]. In the hematopoietic stem cell transplant population, those who received 50 to 60 days of oral ciprofloxacin prophylaxis at 500 mg twice daily demonstrated a decrease in peak BK viruria early after transplantation (range: 7–39 days) and significantly lower rates of hemorrhagic cystitis [13, 14]. The data in the hematopoietic stem cell transplant population suggests that a higher dose of ciprofloxacin may be more effective than what was utilized in our study. A single published report utilizing fluoroquinolone treatment for* de novo* BKV infection in renal transplant recipients demonstrated clinical efficacy [17]. However, a recently reported randomized, double-blind clinical trial found that a 30-day course of levofloxacin did not significantly improve BK viral load reduction or renal allograft function when used in addition to overall reduction of immunosuppression [15]. The patient population in this report differs from ours in one important regard: they received levofloxacin as the initial treatment for BKV infection, whereas our patients received ciprofloxacin after they had failed to clear BK viremia despite immunosuppression reduction and other adjuvant measures. Of note, in our series, we did not find a significant difference in treatment response between those who received ciprofloxacin early (<90 days after the diagnosis of BKV infection) and those who received it later.

Finally, the lack of a control group and the diversity of other anti-BKV therapies that patients had received previously make it difficult to isolate the specific effect of the ciprofloxacin treatment. This point is also noted in the fact that the most pronounced effect was seen with ciprofloxacin in patients who already had improving levels of BK viremia, making it difficult to separate out the effect of ciprofloxacin versus aggressive immunosuppression reduction. Additionally, when we examined the effect of ciprofloxacin by the stage of evolution of BK viremia prior to treatment, we found that a complete clearance of BK viremia at 3 months after treatment with ciprofloxacin was observed in 3 out of 5 patients with low but persistent (<10,000 copies/mL) BK viremia but in 0 out of 4 patients with high BK viremia (>10,000 copies/mL). One possibility is that a longer course of ciprofloxacin is needed to achieve a therapeutic response in this group of patients with high grade BK viremia.

## 5. Conclusions

Ciprofloxacin may be a useful therapeutic tool for BKV infection refractory to conventional treatment. Larger, randomized controlled trials are needed to demonstrate the exact clinical efficacy of ciprofloxacin and other fluoroquinolones in this group of patients. The optimal timing for initiation of treatment, the most efficacious dose and duration of antibiotic treatment, and the patient characteristics and viral subtypes that could potentially predict response to this form of treatment remain to be determined.

## Figures and Tables

**Table 1:** Baseline characteristics.

	Patient 1	Patient 2	Patient 3	Patient 4	Patient 5	Patient 6	Patient 7	Patient 8	Patient 9
Age (years)	66	49	49	74	62	27	43	71	55
Sex	Female	Male	Male	Male	Female	Male	Female	Male	Female
Ethnicity	Asian	African American	African American	Caucasian	Hispanic	Asian	Asian	Hispanic	Asian
ESRD etiology	Hypertension	Hypertension	HIVAN	GN	Drug toxicity	GN	Lupus nephritis	Hypertension	Lupus nephritis
Number of kidney transplants	1	2	1	2	1	1	1	1	1
Donor type	Deceased	Deceased	Deceased	Living unrelated	Deceased	Living related	Deceased	Deceased	Deceased
HLA mismatches	6	5	5	3	5	2	4	6	6
Stent at transplant	No	No	No	No	Yes	No	No	No	No
Induction immunosuppression	Basiliximab	rATG	Basiliximab	rATG	rATG	Basiliximab	Basiliximab	Basiliximab	Basiliximab
Maintenance immunosuppression	MMF, Tac	MMF, Tac, Prd	MMF, CsA, Prd	MMF, Tac, Prd	MMF, Tac, PRD	MMF, Tac, Prd	MMF, Tac, Prd	MMF, Tac	MMF, Tac, Prd
Acute rejection prior to BKV (treatment)	No	No	No	No	No	No	No	Yes (steroids)	No

ESRD: end stage renal disease; HIVAN: human immunodeficiency virus associated nephropathy; GN: glomerulonephritis; HLA: human leukocyte antigen; rATG: rabbit antithymocyte globulin; DGF: delayed graft function; MMF: mycophenolate mofetil; Tac: tacrolimus; Prd: prednisone; CsA: cyclosporine.

**Table 2:** Management of BKV infection prior to addition of ciprofloxacin.

	Patient 1	Patient 2	Patient 3	Patient 4	Patient 5	Patient 6	Patient 7	Patient 8	Patient 9
Time from transplant to BKV diagnosis (days)	91	92	113	224	69	102	31	95	140
SCr at diagnosis (mg/dL)	0.54	1.97	1.27	1.64	1.2	1.58	2.12	1.03	1.08
Viremia at diagnosis (copies/mL)	903	2 ∗ 10^6^	78500	45300	31900	36200	49400	10700	500
Viruria at diagnosis (copies/mL)	4600 ∗ 10^6^	4100 ∗ 10^6^	3000 ∗ 10^6^	1200 ∗ 10^6^	1700 ∗ 10^6^	>500 ∗ 10^6^	116 ∗ 10^6^	170 ∗ 10^6^	5 ∗ 10^6^
Immunosuppression management prior to ciprofloxacin	MMF reduction, Tac to CsA	MMF stopped	MMF stopped, CsA reduction	MMF reduction, Tac reduction	MMF stopped, Tac to CsA	MMF stopped	MMF stopped	MMF reduction, Tac reduction	MMF stopped
Cidofovir prior to ciprofloxacin	No	Yes	Yes	Yes	Yes	Yes	Yes	Yes	Yes
Leflunomide prior to ciprofloxacin	No	No	No	No	Yes	No	No	No	Yes
IVIG prior to ciprofloxacin	No	No	Yes	No	Yes	No	No	Yes	Yes
Time from BKV diagnosis to ciprofloxacin (days)	59	77	130	217	175	199	91	65	379

Abbreviations: BKV: BK polyomavirus; SCr: serum creatinine; IVIG: intravenous immunoglobulin; BKVAN: BK virus associated nephropathy; MMF: mycophenolate mofetil; Tac: tacrolimus; CsA: cyclosporine A.

**Table 3:** Effect of ciprofloxacin on BKV infection.

		Patient 1	Patient 2	Patient 3	Patient 4	Patient 5	Patient 6	Patient 7	Patient 8	Patient 9
Viruria (copies/mL)	Before ciprofloxacin	2 ∗ 10^6^	4600 ∗ 10^6^	110 ∗ 10^6^	14 ∗ 10^6^	1300 ∗ 10^6^	>500 ∗ 10^6^	160 ∗ 10^6^	170 ∗ 10^6^	2 ∗ 10^6^
	Month 3	1015	30 ∗ 10^6^	7 ∗ 10^6^	9 ∗ 10^6^	3100 ∗ 10^6^	>500 ∗ 10^6^	357 ∗ 10^6^	330 ∗ 10^6^	44 ∗ 10^6^
	Clearance	Yes	>50%	>50%	No	No	No	No	No	No

Viremia (copies/mL)	Before ciprofloxacin	830	500	5400	13700	63000	93900	8500	10700	500
	Month 3	0	0	0	1600	167000	41100	4000	7300	3700
	Clearance	Yes	Yes	Yes	>50%	No	>50%	>50%	No	No

BVKAN		Yes	Yes	Yes	No	Yes	No	Yes	Yes	Yes

BKV: BK polyomavirus; BKVAN: BK polyomavirus associated nephropathy.
